# Ovine infectious keratoconjunctivitis in sheep: the farmer’s perspective

**DOI:** 10.1136/vetreco-2018-000321

**Published:** 2019-10-08

**Authors:** Helen J Williams, Jennifer S Duncan, Sarah Nichol Fisher, Amy Coates, Jessica Eleanor Stokes, Amy Gillespie

**Affiliations:** 1 Department of Livestock Health and Welfare, Institute of Veterinary Science, University of Liverpool, Neston, UK; 2 Department of Epidemiology and Population Health, Institute of Infection and Global Health, University of Liverpool, Neston, UK

**Keywords:** sheep, keratoconjunctivitis, eyes, questionnaire, occular disease

## Abstract

The objective of this study was to gather current, farmer-reported data on the frequency of occurrence, risk factors and treatment practices for the sheep eye disease, ovine infectious keratoconjunctivitis (OIKC).

A questionnaire regarding eye disease in sheep was completed by 135 farmers from four livestock markets. Most farmers (87%) had observed OIKC in their flock, 88% of these within the last 2 years.

Farmers reported observing most cases in the winter months (51%) and fewest in the summer (10%). They proposed housing and forage feeding from racks as factors associated with OIKC.

A variety of treatment protocols were used by farmers. The three most popular treatments used were: cloxacillin eye ointment, intramuscular oxytetracycline injection and topical tetracycline spray applied to the eye. Only 62% of treatments were considered very effective by the farmers, with no difference in farmer perceived efficacy between these three most commonly used treatments (p=0.6).

Farmers used 15 different terms to describe a photograph of a sheep with OIKC, including many colloquial terms. We hypothesise that this could result in communication problems between veterinary surgeons and farmers.

## Introduction

Ovine infectious keratoconjunctivitis (OIKC) is an eye disease of sheep. Clinical signs range from mild conjunctivitis to severe keratitis and ulceration, which can result in temporary or permanent blindness. Welfare issues associated with OIKC are the painful nature of the eye condition itself and the impact of blindness on feeding and maternal abilities. It is generally considered to be a common eye disease of sheep and often occurs as a flock level outbreak.^1^ A variety of causative agents have been proposed. *Mycoplasma conjunctivae* is considered the major primary pathogen^2–5^ and clinical disease has been replicated following experimental inoculation.^6 7^ Other pathogens have been implicated but their role is less clear, these include *Staphylococcus aureus*,^6^*Moraxella ovis*,^8 9^*Listeria monocytogenes*,^9^ Chlamydial species^10^ and *Mycoplasma agalactiae*.^11^

There have been few formal epidemiological studies on the frequency of occurrence and risk factors for OIKC infection; however, the disease is believed to be spread between and within flocks through entry of clinically and subclinically infected animals,^3^ facilitated by close contact, for example, at feed troughs.^1 12^

Although a substantial body of evidence exists regarding the aetiology of OIKC, anecdotal evidence from veterinary surgeons and farmers suggests that the disease can be difficult to treat and control both at the individual animal and flock level. Issues frequently raised are that disease recurrence in individual animals post-treatment is common, outbreaks of disease are often prolonged and difficult to contain and there are no established biosecurity protocols to prevent disease spread between flocks.

These problems are thought to occur principally because *M conjunctivae* is known to persist in the conjunctival sac post-treatment, resulting in recurrence of disease in individuals and continued spread of disease to other sheep.^13^ This ability may be a result of biological features of the organism itself or occur as a result of a lack of suitable licensed, efficacious antibiotic treatments. In particular, a lack of drugs that can achieve the necessary bactericidal concentrations in the eye against *M conjunctivae* for a sufficient time to achieve bacteriological cure.

There are only two licensed treatments in the UK available to specifically treat ocular disease in sheep: eye ointment containing cloxacillin (Opticlox; Norbrook, Orbenin Ophthalmic Eye Ointment; Zoetis) which is active against Gram-positive bacteria only and not active against *Mycoplasma* species and an intramuscular injectable preparation of oxytetracycline which is active against a broad spectrum of bacteria as well as *Mycoplasma* species (Terramycin LA; Zoetis and Alamycin LA 300; Norbrook). The route of administration of antibiotics is likely to be a critical factor in treating bacterial eye diseases in order to achieve the necessary inhibitory antibiotic concentrations against the target organisms in the ewe. Indeed, Egwu^14^ demonstrated in vitro sensitivity of *M conjunctivae* to tylosin, oxytetracycline, chlortetracycline and streptomycin, but questioned whether the minimum inhibitory concentration (MIC) of these drugs could be reached for sufficient periods of time in the lacrimal fluid to eliminate the pathogen. The same author^13^ found that although administration of an ocular preparation of chlortetracycline applied once daily for 5 days resulted in clinical cure, *M conjunctivae* was not completely eliminated. Similarly, Hosie and Greig^15^ treated affected lambs with a long-acting injectable preparation of oxytetracycline which resulted in a clinical cure without eliminating the pathogen. More recently, it has been suggested that florfenicol given intramuscularly may be appropriate to treat the condition; however, doses higher than the 20 mg/kg licensed to treat respiratory tract infections are required to reach the MIC for *M conjunctivae* in lacrimal fluid.^16^

Personal communications to the authors of difficulties among vets and farmers in treatment and control OIKC was one of the main drivers for this survey. The objectives of this questionnaire study were to: (1) provide information on farmer reported frequency of occurrence of and risk factors for OIKC; (2) capture farmer reported use of treatments for OIKC and their perceived efficacy and (3) generate hypotheses for research that could improve future management and treatment of this disease.

## Materials and methods

A paper-based questionnaire regarding eye disease in sheep was designed. The questionnaire was composed of 20 questions investigating a range of topics including: demographic data about the farmer and their farm (experience, county and type of area), information about the flock (size, numbers bought in, pedigree status), epidemiological data about eye disease in the flock (presence on farm, seasonality, relation to management), treatment and prevention (online supplementary material).

10.1136/vetreco-2018-000321.supp1 Supplementary data

Four livestock markets situated in England and Wales were visited by the researchers to recruit sheep farmers to participate in the survey. The markets were chosen based on the probability of recruiting farmers from the main sheep farming regions in North Wales, Mid-Wales, North-West England and the South West borders of Scotland. Each market was visited once between the dates of 26 January 2016 and 6 April 2016. A small stand with information about the survey was set up at each location and farmers were directly approached by the researchers, given information about the study verbally and asked if they were interested in participating. Interested farmers were then given a written information sheet and a consent form. Those who consented to participate were given a questionnaire to complete. Although the questionnaire was designed so the farmer could complete it alone, if they asked for assistance (eg, if they had poor eyesight) the researchers would read out the questions and fill in their answers for them. Researchers were also available should the participant want any further clarification. All completed questionnaires were collected on the day of the visit.

As an incentive, participants were given a small bag of sweets on completion of the questionnaire and were also given the option to enter a prize draw in order to win a lambing kit. Personal information was only taken if the farmer wished to enter the prize draw, be involved in future research or receive information regarding the results of the survey. This information was recorded separately to the questionnaire to maintain anonymity.

The data from the completed questionnaires was transcribed into a spreadsheet (Excel 2013; Microsoft) and then imported into Stata V.14 (StataCorp LP) for analysis.

Univariate logistic regression was used to estimate the odds of farmers seeking veterinary advice. Associations tested were: number of breeding ewes categorised in quintiles, whether the disease was mainly seen as an outbreak or individual cases, experience of the farmer and whether they believed their treatment to be effective. The treatments farmers reported using were categorised according to whether a single or multiple form of medication was given. The chi-squared test was used to compare perceived treatment efficacy of a single treatment compared with multiple treatments and to compare perceived treatment efficacy for the three most common treatments.

## Results

### Farmer demographic data

**Figure 1:**
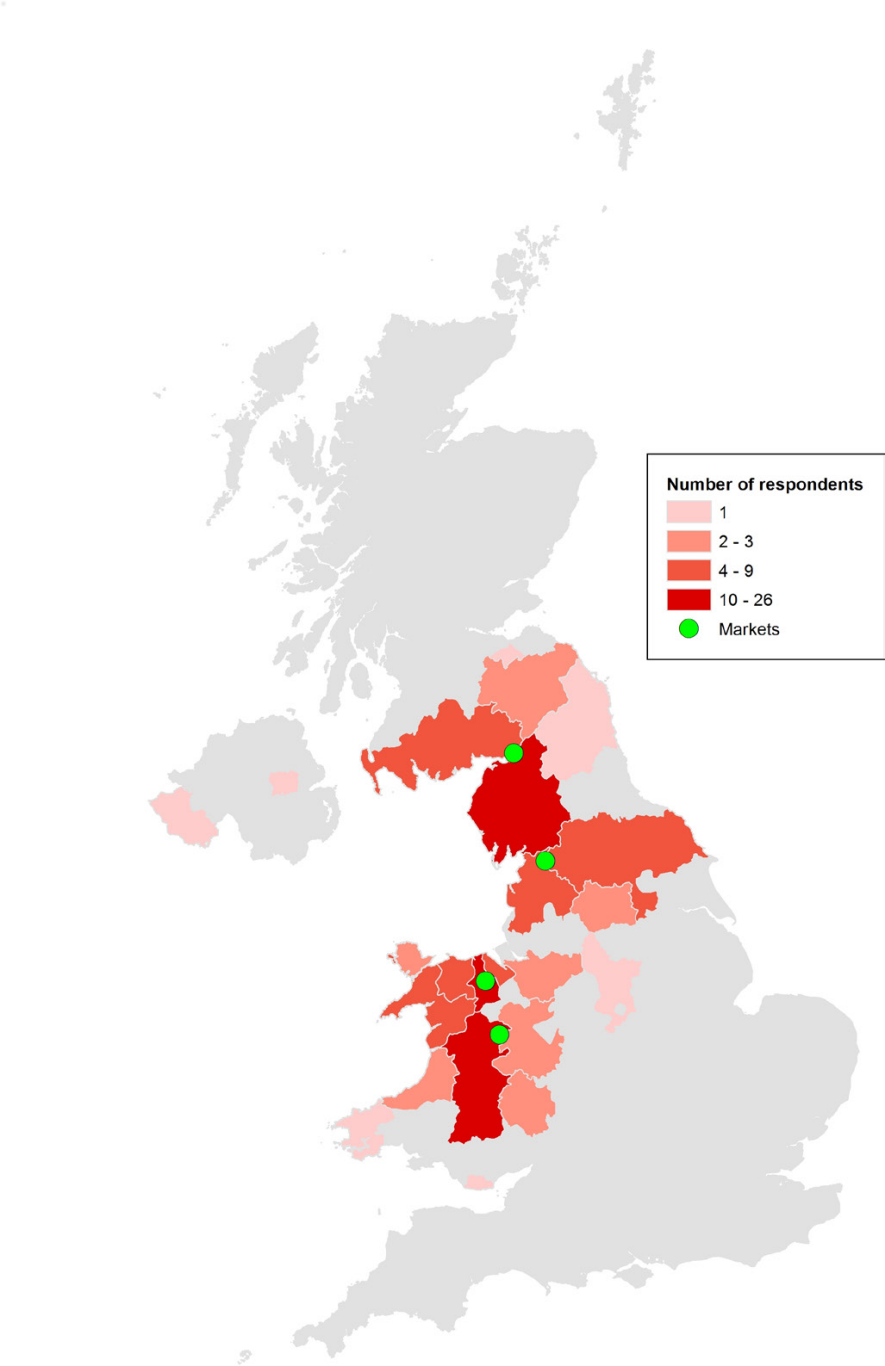
Number of survey respondents by county.

One hundred and thirty-five sheep farmers participated in the study, 42 (31.1%) at market one, 27 (20.0%) at market two, 37 (27.4%) at market three and 29 (21.5%) at market four. The market locations and distribution of the respondents’ farms by county is illustrated in figure 1. Demographic information describing farmer, farm, flock and disease characteristics is shown in table 1.

**Table 1:** Information regarding demographics of participating farmers, farms, flocks and disease characteristics observed by the farmer

Characteristic	Responses (n, %)
*Farmerexperience in years*	**n=135**
≥30	95 (70.4)
20–29	14 (10.4)
10–19	11 (8.1)
6–9	9 (6.4)
1–5	5 (3.7)
*Type of l and*	**n=132**
Hill	30 (22.7)
Upland	55 (41.7)
Lowland	28 (21.2)
Mixed	19 (14.4)
*Flock type*	**n=133**
Commercial	95 (71.4)
Pedigree	6 (4.5)
Mixed	32 (24.1)
*Number of breeding ewes*	**n=135**
Range	0–3000
Median	500
IQR	650
*Respondents purchasing sheep in the last year*	**n=135**
Yes	131 (97.0)
No	4 (3)
*Respondents purchasing rams*	**118** (**87.4**)
Median number purchased	4
Range	1–150
IQR	4
Median number of rams purchased per 100 breeding ewes	0.8
*Respondents purchasing ewes*	**72** (**53.3**)
Median number of ewes purchased	100
Range	4–31 200
IQR	180
Median number of ewes purchased per 100 breeding ewes	15
*Respondents purchasing stores*	**33** (**24.4**)
Median number of stores purchased	300
Range	8–8000
IQR	900
*Disease pattern observed*	**n=112**
Individual animal affected	69 (61.6)
Outbreak affecting multiple animals	43 (38.4)
*Typical percentage affected if seen as an outbreak*	**n=42**
0%–25%	32 (76.2)
26%–50%	6 (14.3)
51%–75%	0 (0)
76%–100%	4 (9.5)
*Category of animal most commonly affected*	**n=116**
Ewes	49 (42.2)
Lambs	17 (14.7)
Ewes and lambs	50 (43.1)

### Farmer reported OIKC epidemiological data

When farmers were shown a picture of ovine infectious keratoconjunctivitis and asked whether they had seen this disease on their farm, 18 (13%) stated they had not seen this disease on their farm and did not participate further. The remaining 117 (87%, 95% CI 80% to 92%) farmers were asked when they had last observed the disease in their flock and 114 responded. The majority (n=78, 68%) had observed OIKC in their flock within the last year, 23 (20%) had seen it between one and 2 years ago and 13 (11%) over 3 years ago. The majority of farmers reported that they saw the disease mainly in individual sheep, rather than as an outbreak (69/112, 61.6%). Farmers who mainly observed outbreaks reported most commonly that 25% or less of the flock were affected (32/42, 76.2%).

One hundred and four farmers responded to a question asking what they called the eye disease shown in the picture of which 89 gave one name for the disease, 14 gave two names and one gave three names, giving a total of 120 responses (table 2).

**Table 2:** Frequency of terms used by 104 farmers to describe OIKC listed by market attended

Name given by farmer for OIKC	Responses (n) (% of responses)	Market1	Market2	Market3	Market4
Newforest/Newforest disease/Newforest eye/Forest eye	45 (37.5%)	17	4	16	8
Cloudy eye/clouded eye/cloudy eye disease	22 (18.3%)	8	2	3	9
Pink eye	17 (14.2%)	6	2	4	5
Snow blindness/snow fever	9 (7.5%)	1	2	3	3
Wind blind/Wind blindness/wind eye/windy eye	5 (4.2%)	0	0	0	5
Bad eye	5 (4.2%)	0	4	1	0
White eye/grey eye	4 (3.3%)	1	2	0	1
Conjunctivitis	4 (3.3%)	0	2	2	0
Silage eye	3 (2.5%)	2	0	1	0
Other (storm eye, misty eye, blindness, fog fever, infection, glazed eye)	6 (5%)	0	2	1	3
Totals	120	35	20	31	34

Similar terms have been grouped for example, snow blindness and snow fever. Eighty-nine farmers gave one name for the disease, 14 gave two names and one gave three names, giving a total of 120 responses.

**Figure 2:**
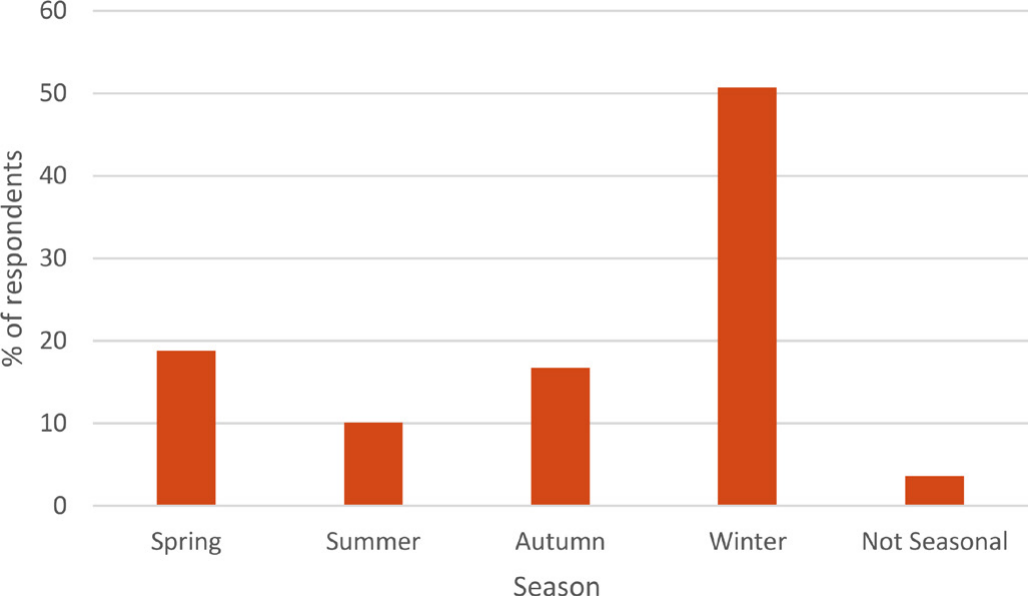
Seasonality of eye disease in sheep reported by farmers.

A question was asked regarding the time of year the disease was most commonly seen, 113 farmers responded. Five stated the disease was not seasonal, 83 chose one season only and 25 chose two seasons (giving a total of 138 answers). Winter was the season most frequently stated to be when the disease was seen (70/138, 50.7%, figure 2).

Farmers identified nine areas of management which they thought were related to occurrence of eye disease (table 3). From 110 respondents, 69 (62.7%) gave one management factor, 19 (17.3%) gave two factors, one (0.9%) gave three factors and 21 (19.1%) stated they could not identify any management factors predisposing to eye disease. Giving a total of 131 responses.

**Table 3:** Management factors reported by 110 farmers as thought to be related to eye disease in sheep

Management factor associated with eye disease	Responses (n)	Percent of responses
Housing	28	21.4
Forage feeding from racks or round bale feeders	21	16.0
Lambing	13	9.9
Bad weather	12	9.2
Buying in	11	8.4
Concentrate feeding or feeding from troughs	9	6.9
Feeding unspecified	7	5.3
Sheep being out/flies	6	4.6
Breed disposition	3	2.3
No management factors identified	21	16.0
Total	131	100%

Sixty-nine farmers suggested one management factor, 19 gave two factors, one gave three factors and 21 could not identify any management factors predisposing to eye disease, giving 131 responses.

### Farmer reported treatment for OIKC

Veterinary advice regarding either diagnosis, treatment or control of eye disease had been sought by 72/117 (61.5%) of farmers. Farmers who saw the disease mainly as an outbreak were more likely to have sought veterinary advice (OR 2.5; 95% CI 1.1 to 5.8, p=0.03). There was no association with number of breeding ewes, farmer experience or how effective they believed the treatment to be (p>0.1)

The majority (n=97/116, 83.6%) stated that they would always treat an individual animal observed with eye disease, 18 (15.5%) answered that they would sometimes treat such an animal and one (0.9%) that they would never treat such an animal. When 43 farmers who stated that they saw the disease as an outbreak were asked if they would only treat the affected animals, or whether they would treat the group, 35 responded as follows: 30 (85.7%) stated they would treat affected animals only, while five (16.7 %) would treat all animals in the group.

Details of the treatments used were reported by 112 farmers (table 4). Five farmers said they would seek veterinary advice before each treatment, 82 farmers gave one form of treatment, while 22 farmers gave each sheep two forms of treatment and three farmers gave each sheep three types of treatment giving a total of 140 responses. The chi-squared test showed no difference in perceived efficacy being ‘very effective’ whether a single form of treatment was administered, or multiple forms of treatment were given to the animal simultaneously (p=0.8).

**Table 4:** Treatments used by farmers for eye disease in sheep

Treatment used	Responses (n)	Percent of responses
Proprietary eye ointment containing cloxacillin	52	37.1
Intramuscular oxytetracycline injection	28	20.0
Tetracycline spray applied topically to the eye	20	14.3
Intramuscular penicillin injection	15	10.7
Antibiotic designed for intramammary use applied topically to the eye	7	5.0
Seek veterinary advice	5	3.6
Intramuscular preparation of oxytetracycline injected subconjunctivally	4	2.9
Intramuscular preparation of penicillin applied topically to the eye	4	2.9
Intramuscular preparation of penicillin injected subconjunctivally	3	2.1
Sugar solution applied topically to the eye	2	1.4
Total	140	100

Eighty-two farmers gave one form of treatment, 22 gave two and three farmers gave each sheep three types of treatment giving a total of 140 responses.

When asked to rate how effective they believed the treatment to be on a scale of 1–4 (1=very effective (75%–100% cure), 2=mostly effective (50%–74% cure), 3=sometimes effective (25%–49% cure) and 4=rarely effective (0%–24% cure)), 106 farmers responded. Sixty-six (62.3%) farmers chose ‘very effective’, 31 (29.3%) ‘mostly effective’, eight (7.6%) ‘sometimes effective’ and one (0.9%) ‘rarely effective’. The three most popular treatments used as a single form of medication were cloxacillin eye ointment, intramuscular oxytetracycline and tetracycline spray applied topically to the eye. The percentage of users reporting these treatments as ‘very effective’ was 62.5%, 66.7% and 53.8%, respectively. The chi-squared test showed no significant difference in perceived effectiveness between these three medications (p=0.60).

## Discussion

The farmers in this survey were not a true random sample of the population of Great Britain (GB) sheep farmers. However, the geographical distribution of farms reflects the density of the sheep population in GB (figure 1).^17^ The farms varied in size and topography, and both the pedigree and commercial sectors were represented. The age category of farmers also reflects the national distribution.^18^ Therefore, we consider the data from the study to be valuable in terms of providing evidence about the frequency of occurrence of OIKC in the UK, the risk factors for disease and data on farmer approaches to its treatment.

In this study, 87% of farmers (95% CI 80% to 92%) stated they had observed eye disease in their flock, 88% of these were within the last 2 years. Therefore, among this study population at least, OIKC remains a common problem.

Farmers reported observing most eye disease cases in the winter months and fewest in the summer. They also perceived housing and forage feeding from racks to be the most important risk factors for OIKC. These findings are in agreement with the anecdotal evidence on OIKC epidemiology.^1 12^ They also suggest the hypothesis that either direct spread from close contact or mechanical damage is more important in the spread of OIKC than vector transmission. This is in contrast to infectious bovine keratoconjunctivitis (IBK) which is most frequently observed during the summer^19^ and whose spread is attributed to the face fly (*Musca autumnalis*),^20^ It is considered that OIKC can be brought into a flock by sheep with mild or inapparent infection.^12^ Since 97% of farmers in this study bought in at least some stock, we were unable to investigate if this was a significant risk factor. Detailed epidemiological studies are required to investigate the hypothesised risk factors for OIKC.

From an animal welfare perspective, a positive finding is that the majority of farmers (84%) stated they would always treat an affected animal. However, a range of different treatments were being administered, many of which were unlicensed for this use. In addition, 39% of farmers stated that they had not ever sought veterinary advice about OIKC, which given the potential severity of the disease for the animal and the flock and the current concerns over responsible antibiotic use is of concern. It is unclear why this is the case, one possibility is lack of perceived importance of eye disease. This is supported by our findings that farmers are more likely to seek veterinary advice for a flock outbreak rather than isolated cases.

Most of the reported treatments were antimicrobials given both topically and systemically. The diverse range of treatments, may reflect a lack of information regarding drug efficacy, as field trials comparing treatments for eye disease are scarce. The majority of farmers (62%) felt that the medication they were giving was effective. However, this does imply that nearly 40% of treatments given are not, which is an obvious concern for the welfare of those animals. A licensed treatment for OIKC, namely, cloxacillin eye ointment or intramuscular oxytetracycline, was reported in 57% of responses. Therefore, use of unlicensed treatments appears to be common and may be regarded as irresponsible antimicrobial use. The safety of such unlicensed use of drugs is unknown and may be a welfare concern, for example, oxytetracycline spray being administered in the eye is likely to be painful.

The most popular treatment was cloxacillin eye ointment, followed by intramuscular oxytetracycline. No difference was detected between these treatments in perceived efficacy by the farmer, despite a previous study demonstrating resistance of *M conjunctivae* to cloxacillin and sensitivity to oxytetracycline when tested by in vitro antibiotic disc sensitivity.^14^ Since no bacteriology was carried out, we do not know which pathogens were responsible for disease observed on these farms. However, as previous studies have reported *M conjunctivae* to be the primary pathogen under UK conditions,^2 4^ it would represent a major change in disease pattern if this pathogen was not present in the majority of cases. Synergism between *M conjunctivae* and both *S aureus*^6^ and *M ovis*^8^ has been demonstrated, resulting in increased severity of clinical signs of OIKC. It is possible that farmers noticed a clinical improvement due to treatment of these pathogens rather than *M conjunctivae*. Another possibility is that OIKC cases may self-resolve, resulting in a high apparent cure rate for all types of treatment.

It is interesting that perceived efficacy was not greater among farmers who used multiple treatments in each sheep, this could indicate that additional medication is being given unnecessarily in these cases.

The results of the survey has raised a number of issues for further research for treatment of OIKC. For example, accurate determination of MIC for the pathogens of interest against licensed antimicrobials coupled with determination of the concentration of active ingredients reached in the lacrimal fluid after treatment, would identify treatments most likely to achieve bacteriological cure. Randomised controlled field trials comparing such treatments would be needed to assess clinical response and produce evidence-based treatment protocols. These would inform individual animal treatment as well as within farm and between farm biosecurity disease prevention protocols.

An interesting finding of this study, and one that would not surprise sheep veterinarians, is that farmers used 15 different terms (which included many colloquial terms) to describe the picture of eye disease presented to them in the study. It is widely recognised that differences in nomenclature and case definition used in scientific publications causes confusion, limits comparison of data between studies and meta-analyses and complicates database searching.^21 22^ Although it is not well documented, it is likely that similar confusion exists due to differences in farmers’ language, for example, lack of repeatable terminology for describing foot lesions in cattle makes comparison of cattle foot-trimming records difficult.^23^ We hypothesise that communication problems caused by using regional dialects may have an impact on animal healthcare. If a veterinary surgeon misunderstands which disease a client is describing, or a farmer is searching for advice on the internet, lack of standard terminology could lead to inappropriate treatment or advice being given. Many farmers used language to describe OIKC that included a risk factor in the title, for example, ‘snow blindness,’ ‘wind blindness’ and ‘silage eye.’ It is possible that this reinforcement of perceived risk factors may cause farmers to be unwilling to adopt new control measures which conflict with their existing beliefs. Qualitative research would help to gauge farmers’ understanding of veterinary terms and how language influences their management and treatment decisions.

This study provides an overview of farmer experience of eye disease in sheep. It has shown that OIKC remains common, raised hypotheses regarding the risk factors for OIKC and demonstrated a need for more efficacious, evidence-based treatment strategies.
