# Supplementary material for: Ovine infectious keratoconjunctivitis in sheep: the farmer’s perspective
Source: Vet Rec Open. 2019 Oct 8;6(1):e000321. doi: 10.1136/vetreco-2018-000321 (PMC6802982; doi:10.1136/vetreco-2018-000321)
Supplement: Supplementary data [file vetreco-2018-000321supp001.pdf]

Eye Disease in Sheep – A questionnaire

About you

1) What County is your farm in?

2) Approximately how long have you been farming for?
(circle answer)

<1 year 1-5years 6-10 years 10-19 years 20-29 years >30 years

About the Flock

3) How many breeding ewes do you have?

4) Is the flock pedigree or commercial?
(circle answer)

Pedigree Commercial A mixture

5) What sort of area is the flock in?
(circle answer)

Hill Upland Lowland

6) Do you buy in sheep and if so how many last year?

Ewes

Rams

Stores

Williams HJ, *et al. Vet Rec Open* 2019; 6:e000321. doi: 10.1136/vetreco-2018-000321

About eye disease in the flock

7) Please take a look at the picture provided, what would you call this eye disease?

8) Have you ever seen this disease on your farm?
(circle answer)

Yes **No**

9) Approximately when did the disease last occur?

10) What group of animals are usually affected (e.g lambs, ewes or both)

11) Does the disease mainly occur in individual sheep or as an outbreak affecting many?

12) If you have had an outbreak roughly what percentage of the flock was affected?

13) What time of year do you think the problem is worst?

14) Does the problem seem to be related to any specific management e.g. housing, tupping, following purchase, lambing etc

15) Have you ever sought veterinary advice about eye problems in the flock (e.g. about treatment, diagnosis, control)

Yes ☐

No ☐

Treatment and control of eye disease

16) If you came across an individual sheep such as the one in the photograph, would you treat it? (circle answer)

Always **Sometimes** **Never**

17) If yes, how would you treat it?

Williams HJ, *et al. Vet Rec Open* 2019; 6:e000321. doi: 10.1136/vetreco-2018-000321

UNIVERSITY OF LIVERPOOL

18) How effective do you find this treatment? (circle answer)

**Very**	**Mostly**	**Sometimes**	**Rarely**
**(75-100% cure)**	**(50-74% cure)**	**(25-49% cure)**	**(0-24%cure)**

19) If you had an outbreak of this disease in the flock affecting many animals would you (circle answer)

a. Treat the affected animals Yes No
b. Treat the whole flock/group Yes No

Please give any further details if relevant.

20) Would you do anything else to try to stop the disease happening or spreading in the flock?

Williams HJ, *et al. Vet Rec Open* 2019; 6:e000321. doi: 10.1136/vetreco-2018-000321
